# Establishing and boosting communication in the European Reference Network for Rare Neurological Diseases (ERN-RND): the impact of offering free educational webinars

**DOI:** 10.1186/s13023-022-02209-9

**Published:** 2022-03-02

**Authors:** Alicia Brunelle Praschberger, Annemarie E. M. Post, Sanja Hermanns, Holm Graessner

**Affiliations:** 1 Institute for Medical Genetics and Applied Genomics, University of Tübingen, Tübingen, Germany; 2 Centre for Rare Diseases, University Hospital Tübingen, Tübingen, Germany

**Keywords:** Rare diseases, Communication strategy, Educational webinars, Social media

## Abstract

**Background:**

Since it first started operating in 2017, the European Reference Network for Rare Neurological Diseases (ERN-RND) implemented a multi-channel communication strategy to effectively reach its target audience: healthcare professionals, patients, researchers, industry representatives and the general public. We first created a website containing useful and up to date information, followed by social media accounts. Here, the analytical data collected about the ERN-RND website and social media channels was compared (Twitter, Facebook, YouTube) during two periods: October 2018 to September 2019, and the year after the ERN-RND free educational webinars were launched: from October 2019 to September 2020. This allowed us to quantify the impact of offering a tangible product (webinars) on the communication strategy.

**Results:**

The analytical data obtained from October 2018 to September 2019 and from October 2019 to September 2020 clearly shows a significant increase in traffic and followers since the launch of the ERN-RND webinars in November 2019. We also created a communication survey which was disseminated between February and June 2021. We collected responses from 61 people: 38 healthcare professionals, 11 scientists, 10 patients (advocates), 2 industry representatives, 1 patient association, 1 charity representative, 1 resident and 1 master student. Most respondents answered “webinars” as the number one reason when asked about which content they look for on the ERN-RND website.

**Conclusions:**

Offering a tangible product—such as the webinars presented in this report—to a specific target group (healthcare professionals) supported our communication strategy by driving traffic to ERN-RND communication channels. It has also successfully tackled ERN-RND’s general aim: by enabling the flow of knowledge on rare neurological and movement disorders to the medical community in hospitals treating patients with these rare and complex conditions, patients ultimately benefit from improved and faster diagnosis, care, and treatment. We aim to set up similar strategies to effectively reach other or the same target groups. For healthcare professionals, organising eConsultations via the Clinical Patient Management System or disseminating standards of care such as diagnostic and therapeutic algorithms as well as clinical practice guidelines might offer potential. For the patient community, organising customised and multilingual webinars could also work.

**Supplementary Information:**

The online version contains supplementary material available at 10.1186/s13023-022-02209-9.

## Background

### Rare diseases

In the European Union, disorders affecting less than 1 in 2000 persons are considered *rare diseases* [1]. More than 8000 different rare diseases affect 30 million people in Europe; around 500,000 of them are affected by rare neurological disorders [2]. While rare diseases are collectively common, each individual one is often only seen in very few patients scattered across different treatment centres, which means that knowledge concerning these disorders is scarce and fragmented across Europe and the world. Consequently, it is difficult for patients to access expert healthcare depending on their EU country of residence and for healthcare professionals to get the most up to date knowledge to diagnose and treat their patients. This is the main challenge that the European Reference Networks (ERNs) are set up to address. They were launched in 2017 by the European Commission as a result of the adoption of Directive 2011/24/EU [3].

### ERN-RND

The European Reference Network for Rare Neurological Diseases (ERN-RND) is a virtual network of 68 specialized healthcare centres located across 24 EU Member states. Its objectives are to improve diagnosis, treatment and care of people affected by low prevalence and complex rare neurological diseases in Europe by sharing knowledge and expertise across borders. The network currently focuses on the following disease groups: Cerebellar Ataxias & Hereditary Spastic Paraplegias; Choreas & Huntington’s Disease; Dystonias, Paroxysmal Disorders & Neurodegeneration with Brain Iron Accumulation (NBIA); Frontotemporal Dementia; Leukoencephalopathies and Atypical Parkinson’s Syndromes.

### ERN-RND communication strategy

As part of its action, the ERN-RND implemented a multi-layered communication strategy focused on several channels. Its aims are to raise awareness for rare neurological diseases (RNDs), collect and share information and knowledge to improve care for RNDs and to build a European RND constituency around ERN-RND. To achieve these aims the communication strategy of the ERN-RND targets a diverse audience composed of patients and patient organisations, healthcare professionals, researchers, policy makers and the general public. To reach this audience, the following communication channels exist: the ERN-RND website, social media channels (Twitter, Facebook, LinkedIn and YouTube), a monthly newsletter and a monthly internal bulletin.

### Aim

In this report we evaluate the online communication strategy of the ERN-RND over the past three years. We compare the results of our communication strategy which were obtained during two periods: from October 2018 to September 2019 and from October 2019 to September 2020, where the ERN-RND started offering free educational webinars in collaboration with the European Reference Network for Rare Neuromuscular Diseases (ERN EURO-NMD) and the European Academy of Neurology (EAN).

## Results

In April 2017, the ERN-RND started its communication activities with the creation of a website. The aim of this website is to create online visibility and to make information about our network and the work we do publicly available. In May 2018, the ERN-RND Twitter account, Facebook account and YouTube channel were created. Here, we analyse the visitor data to our website and social media accounts.

The ERN-RND launched free educational webinars on rare neurological, neuromuscular and movement disorders in November 2019. We started advertising these in October 2019. The aim of the webinars is to share specific knowledge about rare neurological diseases with healthcare professionals; thus filling the gap in the educational landscape and ultimately improving patient care. We compared the usage trends of the ERN-RND website and social media channels between October 2018 and September 2019 to the subsequent year after implementation of the webinars (October 2019–September 2020). Thus, our objective was to assess the impact of the implementation of these webinars on the overall communication reach of ERN-RND. No further communication relevant activities were significantly changed.

**Fig. 1:**
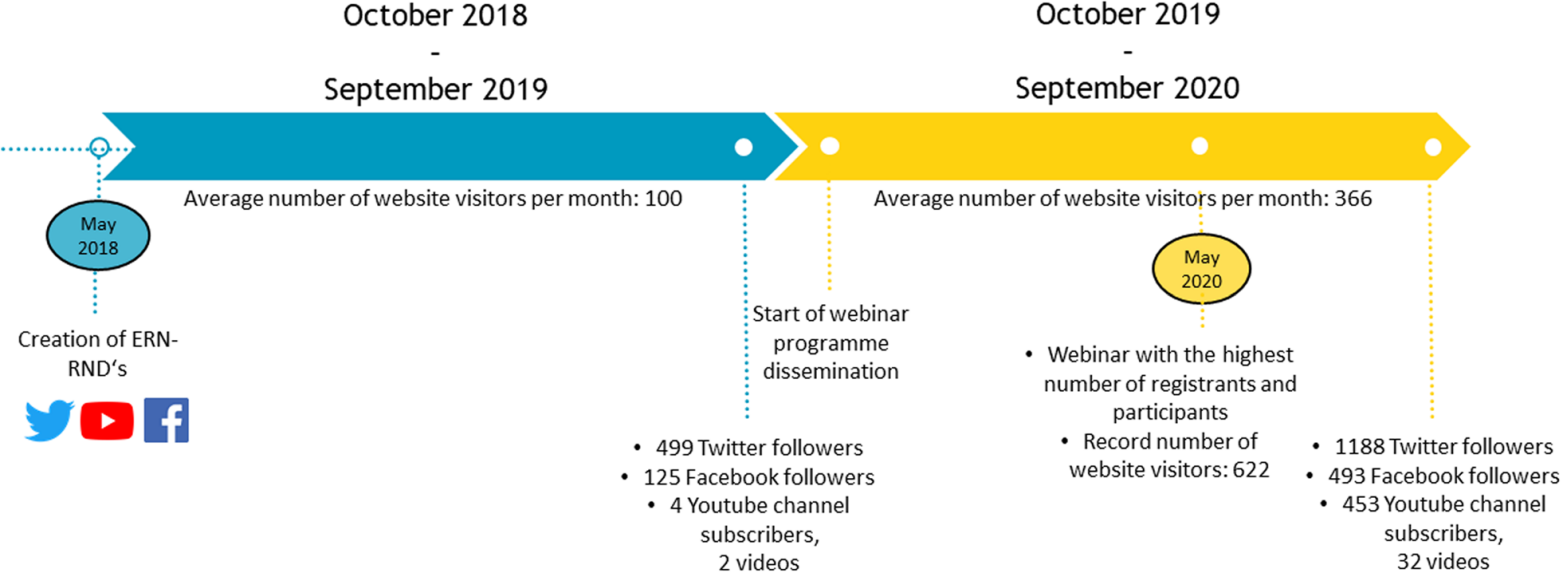
Communication within ERN-RND depicted on a timeline from October 2018 to September 2020

We summarized the two time periods analysed and indicated the different most important communication related events in Fig. 1, using the following indicators of communication reach: monthly website visitors, Twitters followers, Facebook followers, and YouTube subscribers and videos.

### October 2018–September 2019

From 2018 onwards, we started extracting quantitative data relating to traffic to the ERN-RND website [4]. Since then, a total of 8500 visitors from 128 countries visited the ERN-RND website. Between October 2018 and September 2019, 1200 visitors from 64 countries were recorded, averaging to 100 monthly visitors (Fig. 1). During this period, 9289 page views were recorded on the ERN-RND website with the following 5 pages being most frequently visited:

1. ERN-RND homepage: *1750 views*,
2. About us*: 558 views,*
3. Expert centres: *538 views,*
4. The Clinical Patient Management System (previously called “clinicians”): *412 views,*
5. News*: 247 views*

**Fig. 2:**
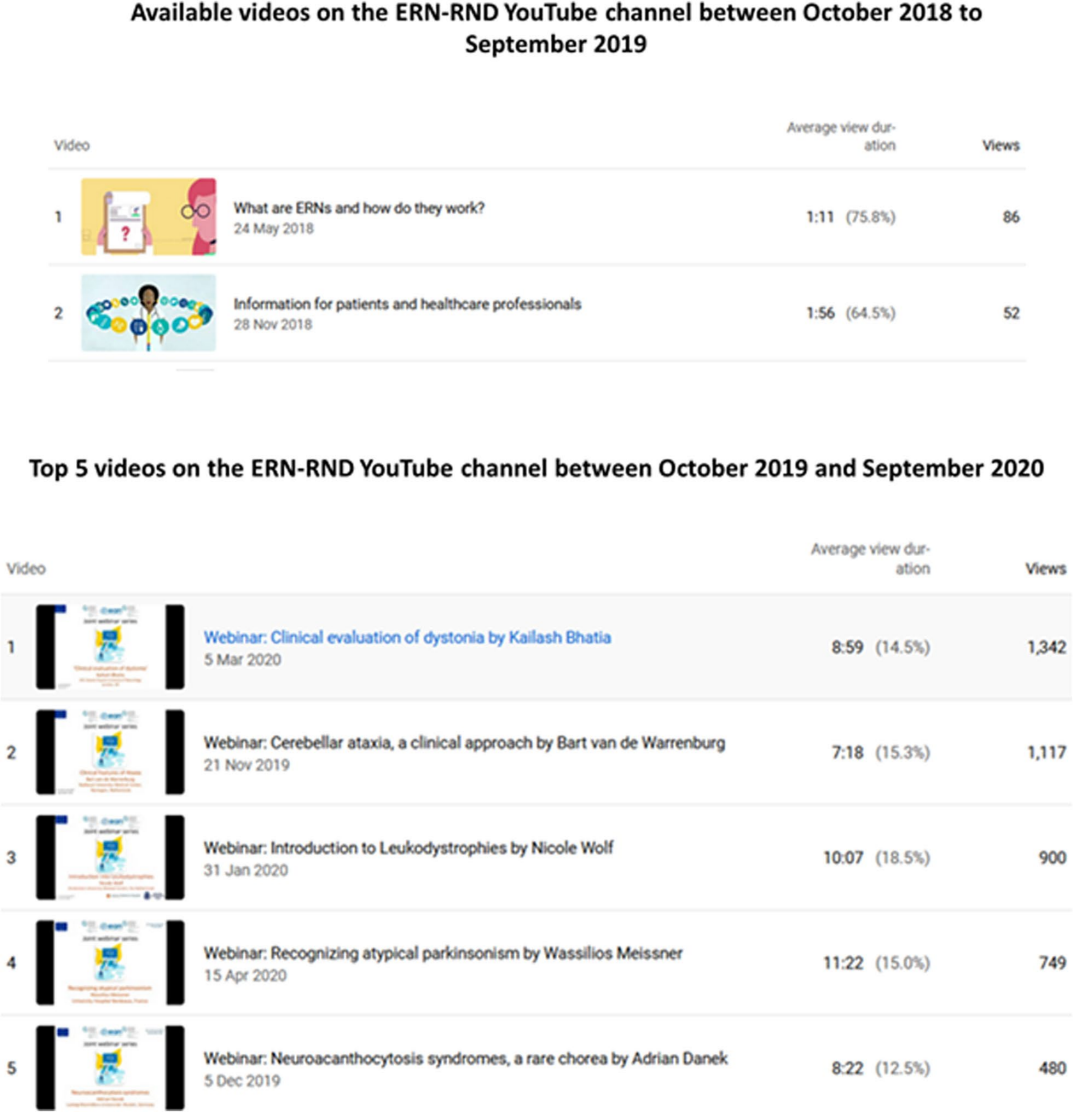
Available videos on the ERN-RND YouTube channel between October 2018 and September 2019; and top 5 videos on the ERN-RND YouTube channel between October 2019 and September 2020

By the end of September 2019, the ERN-RND Twitter account had 499 followers [5], the ERN-RND Facebook account had 125 followers [6] and the YouTube channel had 4 subscribers and 2 videos related to ERN-RND (Fig. 1) [7]. The monthly top tweets between October 2018 and September 2019 are listed in Additional file 1. On YouTube, 138 views were recorded between October 2018 and September 2019 for a total watch time of 3.4 h. The two available videos during this period related to ERN-RND were videos about the European Reference Networks (Fig. 2).

### October 2019–September 2020

In November 2019, the ERN-RND hosted its first educational webinar. We started advertising the ERN-RND webinars around October 2019. Since then, 2–3 webinars per month have taken place on average and 23 webinars were organised in total between October 2019 and September 2020. These are generally presented in English and aimed at clinicians as well as researchers. The patient community is also welcome to attend. Specific information containing the webinar programme and registration links is send through targeted and regular emailing to the clinicians of ERN-RND’s member expert hospitals and staff in charge of communication in relevant institutions such as the European national professional societies for neurology, specific diseases related societies (e.g. ELA—European Leukodystrophy Association—Deutschland, European Huntington Association—EHA), the European Brain Council (EBC), patient organisations (eg. European Federation for Neurological Associations—EFNA), coordination offices of research networks for rare diseases (e.g. Research for Rare- German Networks for Rare Diseases), different institutions active in the rare diseases field (e.g. French Foundation for Rare Diseases—FMR, German Academy for Rare Neurological Diseases—DASNE, Orphanet, Ataxia Global Initiative, Institute imagine France, Ataxia UK, etc.). The information is disseminated through different communication channels and tools that these institutions use. Furthermore, the webinars are announced through the ERN Collaborative Platform (ECP), made available by the European Commission, where they are visible to all ERNs as well as World Wide Neuro which pools together online seminars in the neuroscience field in one website. The European Paediatric Neurology Society (EPNS) actively disseminates relevant information concerning webinars with paediatric focus via their Twitter account and newsletter. Finally, the launch of the webinars on rare neurological, neuromuscular and movement disorders has been a joint initiative between ERN-RND, ERN EURO-NMD and EAN. Hence we support each other in the common communication endeavour to promote the webinars.

To assess the impact of offering these webinars on the reach of our communication activities, we summarize the data from the ERN-RND website and social media accounts here, for the year after our webinar series started. In total, 4400 visitors to our website were recorded between October 2019 and September 2020, which represents an almost fourfold increase in visitors relative to the previous year. The website visitors originated from 111 different countries, which is 47 countries more as compared to the year before. The average number of monthly website visitors increased to 366 during this period. May 2020 was an exceptional month in terms of website visitors, with a record number of 622 unique visitors. This coincides with the most attended webinar on “Paroxysmal dyskinesias: update on clinical and genetic aspects” presented by Giovanna Zorzi on 12 May 2020 which attracted 352 attendees from 64 countries. The total number of page views between October 2019 and September 2020 was 19,801, which represents a two-fold increase compared to the year before. The following 5 webpages were most visited:

1. Education & Training – Webinars *(3380 views)*
2. ERN-RND homepage *(3020 views)*
3. Education & Training – Past webinars *(1515 views)*
4. About us *(619 views)*
5. the Clinical Patient Management System – CPMS *(615 views)*

As of 1 October 2020, the ERN-RND Twitter account had 1188 followers (Fig. 1), with 689 new followers gained between October 2019 and September 2020. On Facebook, the ERN-RND account had 493 followers (Fig. 1), which represents an approximately fourfold increase compared to the previous year. Between October 2019 and September 2020, several of the most popular tweets of the ERN-RND Twitter account were about the ERN-RND webinars (Additional file 2). As of 1 October 2020, the ERN-RND YouTube channel had 453 subscribers (Fig. 1), of which 449 were gained during the last year. 32 videos were displayed, of which 23 were video recordings of the ERN-RND educational webinars. 8112 views were recorded between October 2019 and September 2020 for a total watch time of 1098.3 h. The top 5 videos were recorded webinars and are shown in Fig. 2.

### Communication survey

In order to incorporate direct user feedback in future communication strategy decisions, the ERN-RND conducted a communication survey between February and June 2021 via social media and its newsletter. The survey aimed at collecting the opinions of the ERN-RND stakeholders regarding our general communication strategy. Such information allows us to assess the expectations of our target audience for the different communication channels. In total, 61 responses were collected and analysed. The profiles of the respondents were as follow: 38 healthcare professionals, 11 scientists, 10 patients (advocates), 2 industry representatives, 1 patient association, 1 charity representative, 1 resident and 1 master student.

**Fig. 3:**
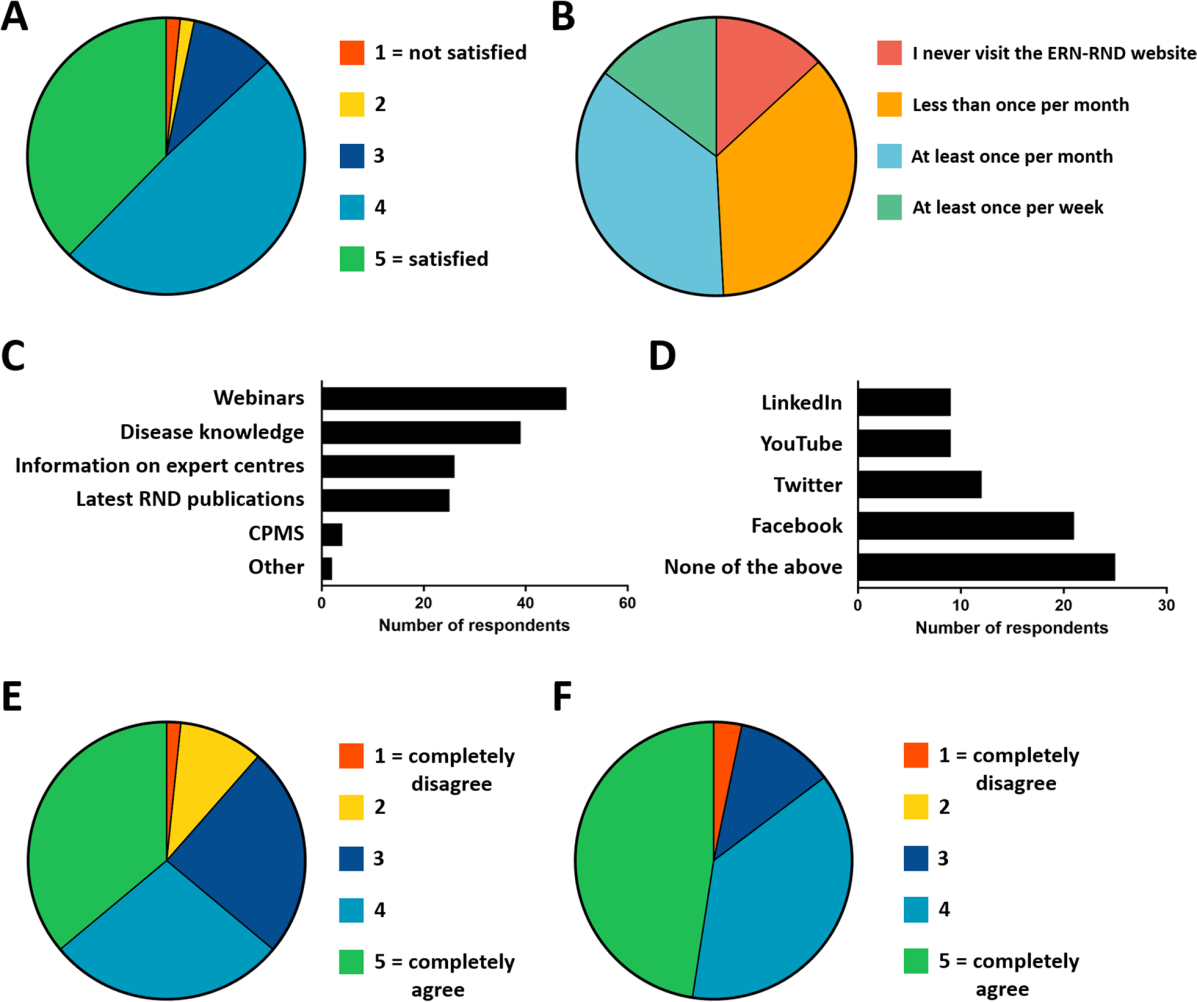
**A** ERN-RND newsletter satisfaction levels, on a scale of 1–5 (1 = not satisfied, 5 = satisfied); **B** Frequency of ERN-RND website visits; **C** Contents the users seek on the ERN-RND website (multiple answers possible); **D** Followed social media accounts; **E** Responses to the statement”ERN-RND is the go-to source for information on rare neurological diseases in Europe” on a scale of 1 to 5 (1 = completely disagree, 5 = completely agree); **F** Responses to the statement”ERN-RND increases awareness of rare neurological diseases” on a scale of 1 to 5 (1 = completely disagree, 5 = completely agree)

We asked the respondents to rate their satisfaction with the information in the newsletter on a scale of 1 (not satisfied) to 5 (satisfied). Of the 61 respondents, 23 were satisfied with the newsletter, while 30 were mostly satisfied (rating 4, Fig. 3A).

2 people indicated they were mostly dissatisfied or dissatisfied (rating 2 and 1, respectively). The remaining 6 respondents were neutral.

When asked which information the respondents would like to receive from the ERN-RND newsletter, answers ranged broadly and included the following:

- disease knowledge,
- webinars,
- interviews with members,
- new trials and drugs,
- latest publications,
- relevant announcements for researchers in the field of RND (seminars, webinars, outstanding papers, scientific events),
- information about funding, and
- best practice of the network (see answers to Question 3).

The survey revealed that 9 respondents visit the website at least once a week, and 22 respondents visit the website at least once per month (Fig. 3B). Another 22 respondents replied that they visit the website less than once per month and 8 people never visit the ERN-RND website.

In relation to the reason of their website visit, respondents were asked for the content they look for when navigating the ERN-RND website. They could choose from multiple answers. The answer the respondents chose most was “Webinars”, followed by “Disease knowledge” (Fig. 3C). “Information on expert centres” and “Latest RND publications” were chosen by 26 and 25 respondents respectively. Only 4 people indicated that they looked for information on the CPMS. 2 respondents indicated that they looked for other information, namely “Figuring out what ERN-RND is all about” and “I have never visited the website”.

Regarding social media, 25 out of 61 respondents answered that they did not follow any of the ERN-RND social media channels (Twitter, Facebook, LinkedIn, YouTube). Out of the four social media accounts, the most frequently followed account was Facebook followed by Twitter (Fig. 3D).

We also asked the survey respondents to rate two important communication objectives: “ERN-RND is the go-to source for information on rare neurological diseases in Europe” (statement 1, Fig. 3E) and “ERN-RND increases awareness of rare neurological diseases” (statement 2, Fig. 3F) from 1 (completely disagree) to 5 (completely agree). For statement 1, 39 respondents indicated that they (completely) agreed, while 7 people (completely) disagreed. 8 people wrote additional comments to statement 1 (Table 1).

**Table 1:** Additional comments from 8 respondents to the statement “ERN-RND is the go-to source for information on rare neurological diseases in Europe”

Comment	Profile
For some rare diseases	Industry representative
Most ERN sites have great easy to follow information	Healthcare professional, patient (advocate)
Zu wenig bekannt und kein Zugang (translation: “not known enough and no access”)	Patient (advocate)
The networks are not generally known yet. Unfortunately	Patient (advocate)
It’s not the main source of information yet, but the awareness is increasing	Patient (advocate)
So many different websites!	Patient (advocate)
I didn’t know	Healthcare professional
As I'm member of Aisa (Ataxia Italian Association) here in Italy, I receive many infos already from them	Patient (advocate)

For statement 2 (“ERN-RND increases awareness of rare neurological diseases”), 52 respondents indicated that they (completely) agreed, while 2 people (completely) disagreed. Additional comments to statement 2 are included in Table 2.

**Table 2:** Additional comments from 8 respondents to the statement “ERN-RND increases awareness of rare neurological diseases”

Comment	Profile
I think we can always do better about raising awareness about healthcare professionals and general public. It's a huge work to do	Scientist
It is difficult to keep all media areas going	Healthcare professional, patient (advocate)
Es ist eine gute Idee (translation: “it is a good idea”)	Patient (advocate)
How could a site and newsletters entirely in English play this role in France?	Patient association
The ERNs are super important not just to raise awareness but to improve access to expertise and improve services	Patient (advocate)
Increasing amount of data presented	Healthcare professional
I think the communication is quite good and being so active also on social media is very good in order to reach the broader RND community	Patient (advocate)
I didn’t know	Healthcare professional

### Twitter poll

**Fig. 4:**
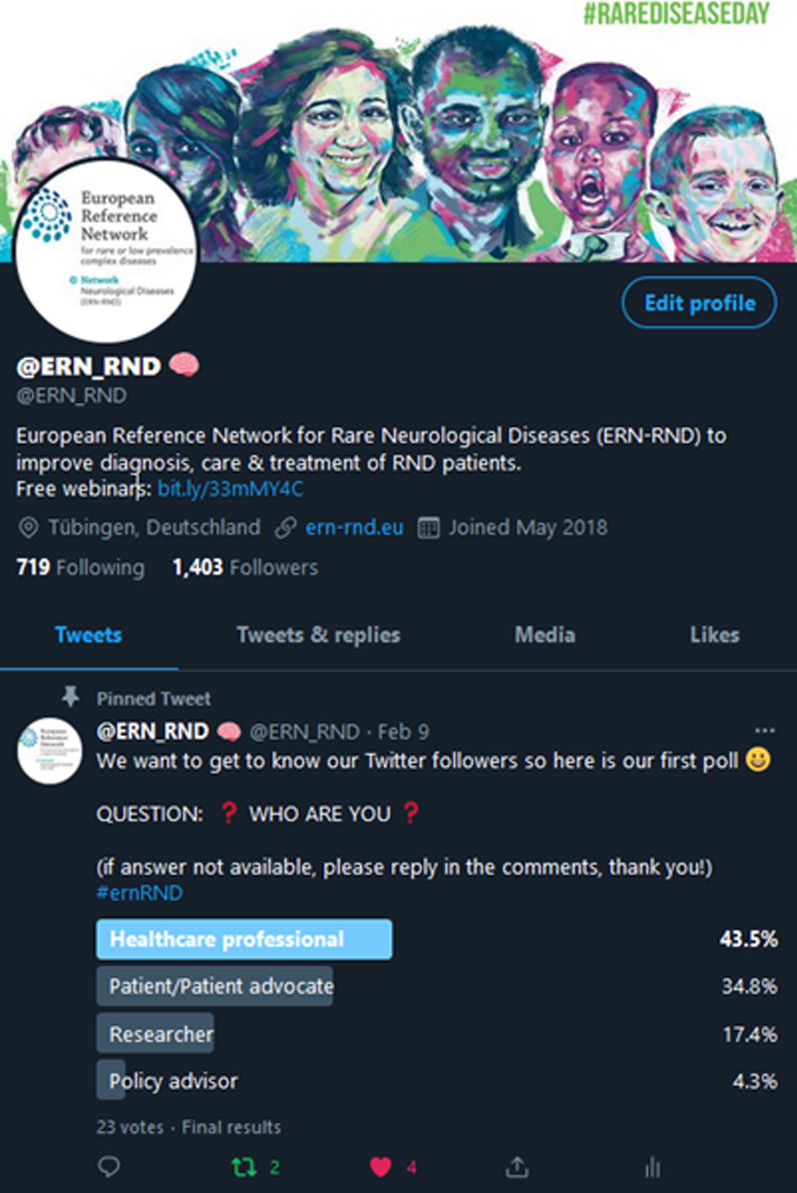
Twitter poll asking followers about their profile

In addition to the communication survey a Twitter poll was carried out, with the aim of better understanding which broad category the ERN-RND Twitter account followers belong to. Overall, 23 responses were recorded. These indicated that most respondents were healthcare professionals (43.5%) followed by patient/patient advocate (34.8%), researcher (17.4%) and policy advisor (4.3%) (Fig. 4).

## Discussion

The first phase of the implementation of the ERN-RND communication strategy between October 2018 and September 2019 saw the creation of the ERN-RND website and social media channels. It was marked by a slow increase in usage of the ERN-RND website and followers on social media channels.

The launch of free educational webinars in collaboration with the European Reference Network for Rare Neuromuscular Disorders (ERN EURO-NMD) and the European Academy of Neurology (EAN) in November 2019 targeted at healthcare professionals focuses on sharing scarce knowledge on rare neurological and movement disorders with our target audience to improve care of patients. Our aim is to fill the gap in the educational offer in rare neurological and movement disorders by making expert knowledge accessible. The webinars have had an important secondary impact onto our communication reach, as it attracted significantly more users to the ERN-RND website, increased the number of followers on social media channels and thus increased the overall visibility of ERN-RND in the global neurological community. The webinars did not only increase our website user base, but also contributed to the wider geographic recognition of ERN-RND, as evidenced by the increase of different countries the website visitors come from.

This clear effect of the implementation of the webinars in 2019 upon website traffic is underlined by the observation that, from this point onwards, the two webinar webpages “webinar programme” and ”past webinars” were regularly amongst the top two most visited webpages of the ERN-RND website. The webpage “Education & Training—Webinars” replacing “ERN-RND homepage” as the top-ranked page is a further indication of the significant effect of the implementation of the webinars upon the ERN-RND overall website traffic. Furthermore, the record number of visitors to the ERN-RND website in May 2020 could be explained by the organisation of two webinars, one of them being the most attended since the launch.

Between October 2019 and September 2020, the most popular social media posts were consistently about the ERN-RND webinars. In addition to this, the number of subscribers to the ERN-RND YouTube channel increased 11-fold in only 6 months (April–September 2020), likely as a consequence of ERN-RND webinar recordings being stored there so that interested viewers can re-watch most of the webinars on-demand. The number of views and cumulative watch time of ERN-RND YouTube videos increased several-fold between October 2019 and September 2020.

Joining forces with well-known organisations such as the EAN, the ERN EURO-NMD and the EPNS greatly boosted the promotion of our implemented webinars and enabled us to reach a large audience spread across Europe and even beyond. Indeed, by advertising the webinars, they helped to increase visibility of ERN-RND amongst our target audience as well as gain credibility as an important source—increasingly the go-to source—of information and knowledge in the field of rare neurological diseases. This also allowed us to strengthen these relationships, which is very important as they are allies in our mission to support patients with RNDs in Europe. Taken together, the sharp increase in ERN-RND website and social media activity upon implementation of webinars centred around the diagnosis and treatment of RNDs clearly shows that providing a tangible product not only provides specific value to the target audience but significantly improved ERN-RND visibility to all major stakeholders, moreover, covering the full activity spectrum of the network.

The results of the communication survey were useful to provide evidence that the ERN-RND communication strategy works. Not only did the survey give confirmation on the perceived high quality of our communication content but it also provided information on the regular usage and high attractivity of our communication channels. Based on the responses that shows a recognition of the quality of the communication content, a regular usage pattern and a focus on RND knowledge access, the implemented communication strategy clearly contributes to achieving the ERN-RND goal to provide the most up to date knowledge to diagnose and treat RND patients to healthcare professionals.

The results of the survey will inform the future ERN-RND communication strategy by taking the remarks of the respondents into account. It will help, for example, in providing a wider variety of content to our audience such as new trials and drugs. It also helps to confirm that the information already provided, e.g. on the ERN-RND website, is useful to our target audience. We aim to set up similar strategies to effectively reach other or the same target groups. For healthcare professionals, organising eConsultations via the Clinical Patient Management System (CPMS) or disseminating standards of care such as diagnostic and therapeutic algorithms as well as clinical practice guidelines might offer potential. For the patient community, customised and multilingual webinars could also achieve similar results.

Furthermore, the results of the communication survey reinforce the impact of the webinars, since most respondents answered that they look for information on webinars when visiting the ERN-RND website. This strengthens our key argument that offering a tangible service, such as webinars, can increase the visibility, awareness and impact of a network as well as drive traffic to websites and social media accounts. In other words, in addition to providing valuable RND specific knowledge to its viewers, it has an important secondary benefit in supporting the overall communication strategy as well as help the network to achieve its aims.

Our aim is to improve the webinars further. To this end, we gather feedback from presenters and participants. Participant feedback is collected through a post-webinar survey, where we ask participants to rate the webinar and tell us how relevant it was for them. Some past suggestions by attendees are the use of patient videos to illustrate particular conditions, a clear and structured way of presenting with defined educational outcomes, and embedding the theory presented in a case study. We highlight the usefulness of these methods to new webinar speakers in the preparation phase. So far, many webinar speakers have implemented these suggestions in their presentations.

In future, the webinar strategy of ERN-RND will pursue two approaches. Firstly, we will be offering recurrent disease (group) specific webinars which serve the purpose of providing practical and state- of-the-art knowledge to treat patients. Secondly, we have introduced new webinar foci such as “advanced therapies” and “clinical trials” to stay up to date with new developments in the field. We will sustain the webinar series and include it in the postgraduate curriculum that ERN-RND is developing with the ERN EURO-NMD) and the European Reference Network for rare epilepsies (EpiCARE).

## Conclusions

Offering a tangible product—such as the webinars presented in this report—to a specific target group (healthcare professionals) supported our communication strategy by driving traffic to ERN-RND communication channels. It has also successfully tackled ERN-RND’s general aim: by enabling the flow of knowledge on rare neurological and movement disorders to the medical community in hospitals treating patients with these rare and complex conditions, patients ultimately benefit from improved and faster diagnosis, care, and treatment. We aim to set up similar strategies to effectively reach other or the same target groups. For healthcare professionals, organising eConsultations via the Clinical Patient Management System (CPMS) or disseminating standards of care such as diagnostic and therapeutic algorithms as well as clinical practice guidelines might offer potential. For the patient community, customised and multilingual webinars could also achieve similar results.

## Methods

We analysed data regarding visits to the ERN-RND website and activity on our social media channels. The communication channels used to disseminate information (including ERN-RND webinars) to our target audience are the following:

**Table:** 

Communication channels ERN-RND	Link
Website	www.ern-rnd.eu
Twitter (social media)	https://twitter.com/ERN_RND
Facebook (social media)	https://www.facebook.com/ErnRnd-638837783116369/
LinkedIn (social media)*	https://www.linkedin.com/company/ern-rnd
YouTube	https://www.youtube.com/channel/UCLpEdEyhGnQpdmLLzqNXkTg
Newsletter**	http://www.ern-rnd.eu/news/newsletters/
Bulletin (internal newsletter)***	Not available

*Traffic to the ERN-RND LinkedIn page was not included in this analysis, because of its recent start date (July 2020)

**Data about the ERN-RND Newsletter was not included in this analysis because of the lack of precise data

***Data about the ERN-RND Bulletin was not included in this analysis because of its recent start and lack of available data during the studied time period

### Evaluation of website traffic

Google Analytics was used to track user behaviour on the ERN-RND website. We evaluated the following parameters: number of users, total page views including single page view breakdown, and the geographical location of users.

### Evaluation of social media activity

Data about ERN-RND social media’s activity was directly extracted from the social media channels themselves: Twitter, Facebook and YouTube. The top videos on YouTube were selected according to average view durations and number of views. For Twitter, we looked at the number of followers and top tweets. These last ones were determined by the number of impressions a tweet receives, the number of engagements and the engagement rate. As for Facebook, we compared the number of followers over time.

### Communication survey

We analysed our target audience and their expectations of ERN-RND’s online activities via a communication survey. We composed ten open-ended as well as close-ended questions written in English (Additional file 3). The survey was first sent to all ERN-RND Newsletter recipients, including ERN-RND members, in February 2021. To increase the number of respondents, we included it again in the April/May/June 2021 Newsletters as well as shared it via the ERN-RND Twitter and Facebook channels.

### Twitter poll

A Twitter poll was shared on the ERN-RND Twitter account on 9 February 2021 for a duration of 7 days asking the audience who they were. 23 people/accounts responded.

## Supplementary Information

**Additional file 1**. Monthly top tweets between October 2018 and September 2019

**Additional file 2**. Monthly top tweets between October 2019 and September 2020

**Additional file 3**. ERN-RND communication strategy survey. The survey that was distributed to ERN-RND members and patient representatives, ERN-RND Newsletter subscribers as well as via ERN-RND social media channels

## Data Availability

Not applicable.

## Abbreviations

ERNs: European Reference Networks
ERN-RND: European Reference Network for Rare Neurological Diseases
RNDs: Rare Neurological Diseases
ERN EURO-NMD: European Reference Network for Rare Neuromuscular Diseases
EAN: European Academy of Neurology
ELA Deutschland: European Leukodystrophy Association Deutschland
EHA: European Huntington Association
EBC: European Brain Council
EFNA: European Federation of Neurological Associations
DASNE: Deutsche Akademie für Seltene Neurologische Erkrankungen (German Academy of Rare Neurological Diseases)
ECP: ERN Collaborative Platform
EPNS: European Paediatric Neurology Society
CPMS: Clinical Patient Management System
