# Supplementary material for: Establishing and boosting communication in the European Reference Network for Rare Neurological Diseases (ERN-RND): the impact of offering free educational webinars
Source: Orphanet J Rare Dis. 2022 Mar 2;17:89. doi: 10.1186/s13023-022-02209-9 (PMC8889675; doi:10.1186/s13023-022-02209-9)
Supplement: Supplementary file 1 — Additional file 1. Monthly top tweets between October 2018 and September 2019 [file 13023_2022_2209_MOESM1_ESM.pdf]

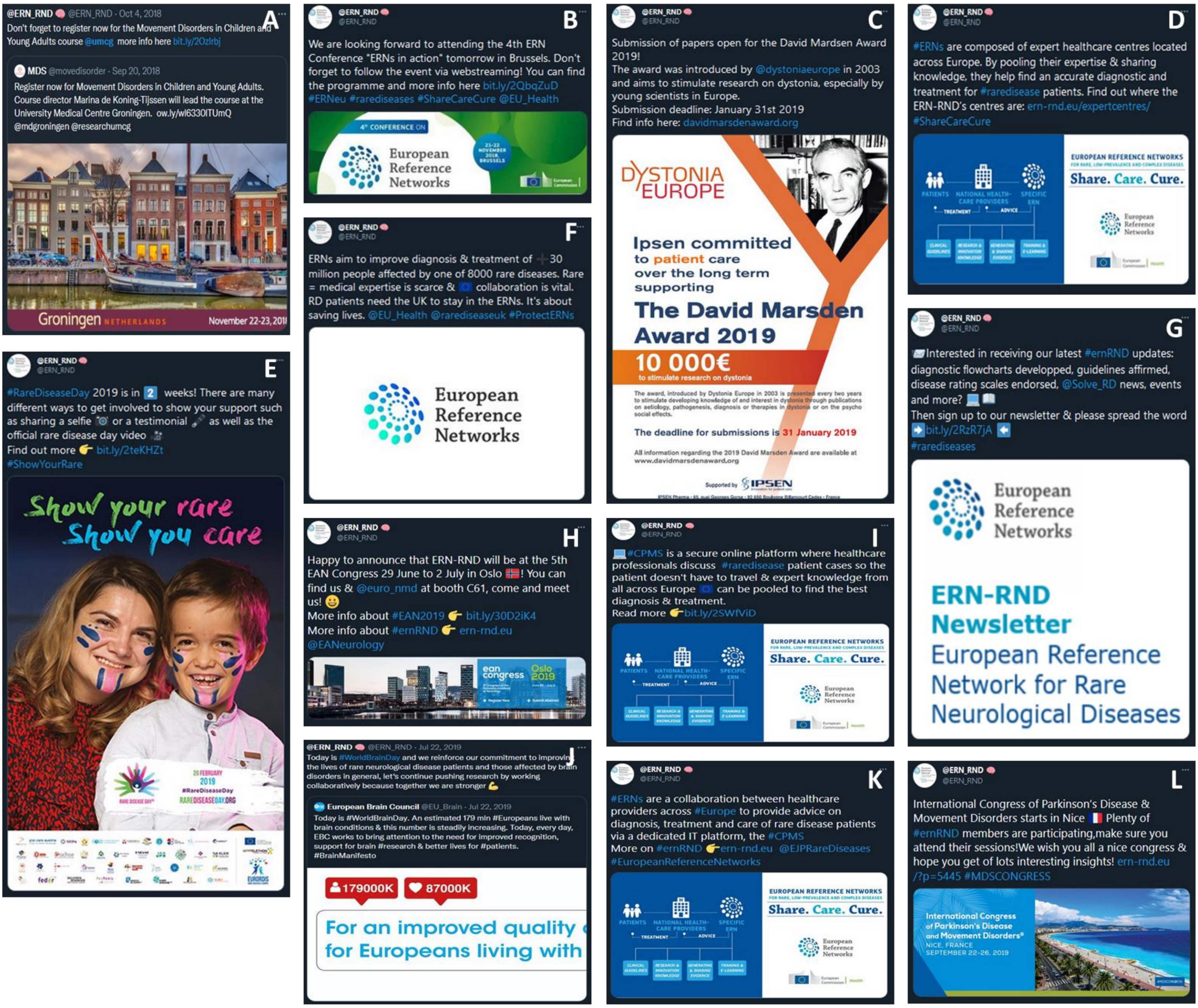

Additional file 1. Top tweets between October 2018 and September 2019.

A) Retweet from the Movement Disorders Society about a course (1,391 impressions; 11 total engagements; 6 likes; 1 retweet; 4 October 2018); **B)** Fourth ERN Conference in Brussels (5,905 impressions; 41 total engagements; 10 likes; 8 retweets; 20 November 2018); **C)** Paper submission for the David Marsden Award (1,159 impressions; 27 total engagements; 7 likes; 4 retweets; 6 December 2018); **D)** ERN-RND expert centres (11,081 impressions; 211 total engagements; 20 likes; 11 retweets; 14 January 2019); **E)** Rare Disease Day 2019 (1,973 impressions; 16 total engagements, 5 likes; 2 retweets; 14 February 2019); **F)** UK staying in the ERNs (5,391 impressions; 56 total engagements; 20 likes; 13 retweets; 25 March 2019); **G)** ERN-RND Newsletter (4,720 impressions; 28 total engagements; 10 likes; 5 retweets; 8 April 2019); **H)** ERN-RND at the EAN Congress 2019 (2,367 impressions; 23 total engagements; 7 likes; 5 retweets; 23 May 2019); **I)** Clinical Patient Management System – CPMS (3,813 impressions; 23 total engagements; 7 likes; 7 retweets; 5 June 2019); **J)** Retweet from the European Brain Council for World Brain Day (4,458 impressions; 22 total engagements; 13 likes; 5 retweets; 22 July 2019); **K)** European Reference Networks – ERNs (2,158 impressions; 24 total engagements; 8 likes; 5 retweets; 19 August 2019); **L)** International Congress of Parkinson's Disease and Movement Disorders 2019 (2,139 impressions; 14 total engagements; 9 likes; 22 September 2019)
