# Supplementary material for: Establishing and boosting communication in the European Reference Network for Rare Neurological Diseases (ERN-RND): the impact of offering free educational webinars
Source: Orphanet J Rare Dis. 2022 Mar 2;17:89. doi: 10.1186/s13023-022-02209-9 (PMC8889675; doi:10.1186/s13023-022-02209-9)
Supplement: Supplementary file 2 — Additional file 2. Monthly top tweets between October 2019 and September 2020 [file 13023_2022_2209_MOESM2_ESM.pdf]

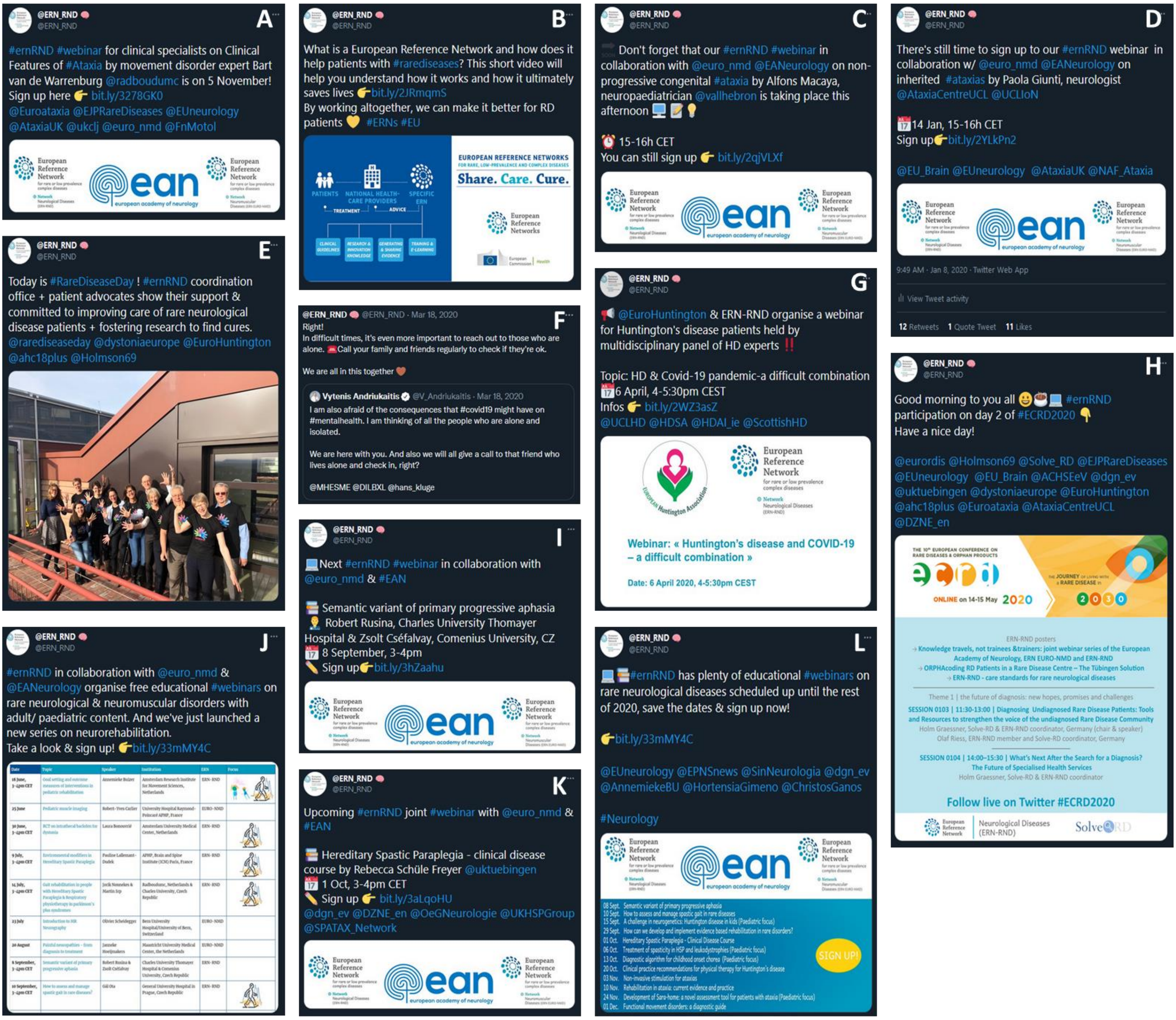

Additional file 2. Top tweets between October 2019 and September 2020.

A) Announcing ERN-RND webinar on 'clinical features of ataxia' (2,584 impressions; 82 total engagements; 17 likes; 11 retweets; 14 October 2019); **B)** Video about ERNs (3,282 impressions; 48 total engagements; 11 likes; 14 retweets; 26 November 2019); **C)** Announcing ERN-RND webinar on 'non-progressive congenital ataxia' (4,739 impressions; 29 total engagements; 7 likes; 5 retweets; 17 December 2019); **D)** Announcing ERN-RND webinar on 'inherited ataxias' (3,989 impressions; 57 total engagements; 11 likes; 13 retweets; 8 January 2020); **E)** Rare Disease Day 2020 (2,747 impressions; 77 total engagements; 28 likes; 13 retweets; 29 February 2020); **F)** Retweet from Vytenis Andriukaitis on consequences of COVID-19 on mental health (8,502 impressions; 20 total engagements; 9 likes; 6 retweets); **G)** Announcing European Huntington Association and ERN-RND webinar on 'Huntington's disease and COVID-19 – a difficult combination' (8,042 impressions; 121 total engagements; 13 retweets; 13 likes; 1 April 2020); **H)** ERN-RND at the European Conference on Rare Diseases and Orphan Products 2020 – ECRD (3,765 impressions; 36 total engagements; 10 likes; 15 May 2020); **I)** Announcing ERN-RND webinar on 'semantic variant of primary progressive aphasia' (2,106 impressions; 67 total engagements; 9 likes; 6 retweets; 16 July 2020); **J)** Announcing ERN-RND series of webinars on neurorehabilitation (2,629 impressions; 137 total engagements; 18 likes; 15 retweets; 8 June 2020); **K)** Announcing ERN-RND webinar on 'Hereditary Spastic Paraplegia – clinical disease course' (2,528 impressions; 82 total engagements; 7 likes; 9 retweets; 31 August 2020); **L)** Announcing end of 2020 ERN-RND webinar programme (4,982 impressions; 560 total engagements; 31 likes; 31 retweets; 4 September 2020)
