# Supplementary material for: Establishing and boosting communication in the European Reference Network for Rare Neurological Diseases (ERN-RND): the impact of offering free educational webinars
Source: Orphanet J Rare Dis. 2022 Mar 2;17:89. doi: 10.1186/s13023-022-02209-9 (PMC8889675; doi:10.1186/s13023-022-02209-9)
Supplement: Supplementary file 3 — Additional file 3. ERN-RND communication strategy survey. The survey that was distributed to ERN-RND members and patient representatives, ERN-RND Newsletter subscribers as well as via ERN-RND social media channels [file 13023_2022_2209_MOESM3_ESM.pdf]

Questions

To which of the following groups do you belong?

- Healthcare professional
- Scientist
- Patient (advocate)
- Industry representative
- Policy advisor
- Patient association
- Master Student
- Charity representative
- Resident

How satisfied are you with the information shared via the newsletter?

Rank from 1 (completely dissatisfied) - 5 (completely satisfied)

What information would you like ERN-RND to share with you?

…….

How often do you visit the ERN-RND website?

- At least 1x per week
- At least 1x per month
- Less than 1x per month
- I never visit the ERN-RND website

What do you look for on the ERN-RND website?

- Information on expert centres
- Disease knowledge
- CPMS
- Webinars
- Latest RND publications
- Other …

Which of the ERN-RND social media accounts do you follow?

- Twitter
- Facebook
- LinkedIn

- YouTube
- None of the above

What is your opinion on the following statement: "ERN-RND is the go-to source for information on rare neurological diseases in Europe"?

Rank from 1 (completely disagree) - 5 (completely agree)

Do you want to add a comment on this statement?

……….

What is your opinion on the following statement: "ERN-RND increases awareness of rare neurological diseases"?

Rank from 1 (completely disagree) - 5 (completely agree)

Do you want to add a comment on this statement?

……….
